# MiR-3662 suppresses hepatocellular carcinoma growth through inhibition of HIF-1α-mediated Warburg effect

**DOI:** 10.1038/s41419-018-0616-8

**Published:** 2018-05-10

**Authors:** Zhiqiang Chen, Xueliang Zuo, Yao Zhang, Guoyong Han, Long Zhang, Jindao Wu, Xuehao Wang

**Affiliations:** 1 Hepatobiliary Center, The First Affiliated Hospital of Nanjing Medical University, Key Laboratory of Liver Transplantation, Chinese Academy of Medical Sciences, Nanjing, 210029 China; 2 Department of Gastrointestinal Surgery, The First Affiliated Hospital, Yijishan Hospital of Wannan Medical College, Wuhu, 241001 China; 3 State Key Laboratory of Reproductive Medicine, Nanjing Medical University, Nanjing, 210029 China

## Abstract

Glucose metabolic reprogramming from oxidative to aerobic glycolysis, referred as the Warburg effect, is a hallmark of tumor cells. Accumulating evidence suggests that a subset of microRNAs play pivotal roles in modulating such reprogramming of glucose metabolism in cancer cells. miR-3662 has been implicated previously in both pro-tumorigenic and anti-tumorigenic effects in several types of cancer. The expression level of miR-3662 is downregulated in acute myeloid leukemia, whereas increased miR-3662 expression is observed in lung adenocarcinoma. However, the roles and underlying mechanisms of miR-3662 in hepatocellular carcinoma (HCC) metabolic reprogramming remain unclear. Our present study revealed that miR-3662 was frequently downregulated in HCC tissues and cell lines. The low expression level of miR-3662 was associated with tumor size, tumor multiplicity, Edmondson grade, and tumor-node-metastasis stage. Gain-of-function and loss-of-function assays showed that miR-3662 dampened glycolysis by reducing lactate production, glucose consumption, cellular glucose-6-phosphate level, ATP generation, and extracellular acidification rate, and increasing oxygen consumption rate in HCC cells after treatment with the hypoxia mimetic CoCl_2_. Moreover, miR-3662 suppressed cell growth *in vitro* and *in vivo*, and induced G1/S cell cycle arrest. miR-3662 inhibited the activation of ERK and JNK signaling pathways in HCC. By combined computational and experimental approaches, hypoxia-inducible factor-1α (HIF-1α) was determined as a direct target of miR-3662. After treatment with the hypoxia mimetic CoCl_2_, miR-3662 regulated the Warburg effect and HCC progression via decreasing HIF-1α expression. Our findings uncover a mechanistic role for miR-3662/HIF-1α axis in HCC metabolic reprogramming, providing a potential therapeutic strategy in liver cancer.

## Introduction

Hepatocellular carcinoma (HCC) is the second leading cause of cancer-related mortality worldwide, with ~782,500 new cases occurring each year^1,2^. Although many therapeutic strategies, such as surgical resection, liver transplantation, and radiofrequency ablation, have been employed, its prognosis remains unfavorable^3,4^. Given the asymptomatic nature of this disease, most patients are diagnosed at advanced stages. In this regard, elucidating the molecular processes and mechanisms underlying liver cancer might have a significant bearing on the systematic treatment of HCC.

Metabolic alteration is a typical hallmark of cancer cells^5^. Despite adequate availability of oxygen, tumor cells tend to generate energy from aerobic glycolysis rather than depend on mitochondrial oxidative phosphorylation. This phenomenon, defined as the Warburg effect, often results in increased glucose uptake, accumulation of ATP, and lactate production in tumor cells^6^. Although glycolysis generates less ATP than oxidative phosphorylation, the Warburg effect confers advantages to cell growth not only by providing the carbon sources that are required for rapid cell proliferation but also by minimizing the production of reactive oxygen species^7^. Normal differentiated hepatocytes do not generate energy from aerobic glycolysis under non-hypoxic conditions. In HCC, however, extensive reprogramming of metabolic pathways occurs^7,8^. Thus, the understanding of this process is essential to identify novel targets for HCC therapy.

MicroRNAs (miRNAs), a class of small noncoding RNAs composed of ~22 nucleotides, function as posttranscriptional regulators by inducing degradation of mRNAs of target genes. Targeting most protein-coding transcripts, miRNAs are involved in almost all developmental and pathological processes in humans^9^. Emerging studies have revealed the pivotal role of miRNAs in cancer cell metabolism^10^. The underlying mechanisms by which miRNAs reprogram cancer metabolism, however, are still largely unknown. Recently, it was found that miR-3662 is upregulated during hematopoietic differentiation, whereas its expression is downregulated in acute myeloid leukemia^11^. Increased expression level of miR-3662 is observed in lung adenocarcinoma^12,13^. Yet the association of miR-3662 with cellular metabolism regulation in HCC has not been investigated.

In the current study, we identified that miR-3662 expression was markedly reduced in HCC tissues and cell lines. Also, miR-3662 suppressed the glycolytic pathway, cellular proliferation, and induced cell cycle arrest in liver cancer cells after treatment with the hypoxia mimetic CoCl_2_. ERK and JNK signaling pathways were regulated by miR-3662. Mechanistically, hypoxia-inducible factor-1α (HIF-1α) was a novel target of miR-3662. By directly targeting HIF-1α, miR-3662 suppressed liver cancer cell glycolysis and proliferation. These findings indicated that the miR-3662/HIF-1α axis was highly correlated with the malignant phenotypes and modulated the reprogramming of glucose metabolism in HCC.

## Methods and materials

### Cell lines and human tissues

Human noncancerous hepatic cell lines (LO2 and QSG-7701) and HCC cell line SMMC7721 were obtained from American Type Culture Collection (Rockville, MD, USA). Hep3B, HepG2, Huh7, and HCCLM3 cells were purchased from Shanghai Institute of Cell Biology, Chinese Academy of Sciences (Shanghai, China). We isolated primary human hepatocytes from liver resections of consenting patients undergoing partial hepatectomy, using a modified two-step collagenase perfusion technique, as previously described^14^. All cell lines were maintained in DMEM (Gibco, Carlsbad, CA, USA) supplemented with 10% fetal bovine serum and 1% penicillin-streptomycin within a humidified incubator containing 5% CO_2_ at 37 °C. We used cobalt chloride (CoCl_2_; Sigma-Aldrich, St Louis, MO, USA) to simulate hypoxic conditions as previously reported^15^. Briefly, cells were treated with 150 μM CoCl_2_ and incubated for 48 h. HCC samples (*n* = 50) were acquired from consenting patients undergoing hepatic resection in the First Affiliated Hospital of Nanjing Medical University. Experiments were reviewed and approved by the Ethics Committee of the First Affiliated Hospital of Nanjing Medical University.

### RNA extraction, reverse transcription, and real-time quantitative PCR

Total RNA was extracted from cells treated with hypoxia-mimetic agent CoCl_2_ and tissue samples using TRIzol reagent (Invitrogen, Carlsbad, CA, USA) according to the manufacturer’s protocol. Reverse transcription was preformed using the PrimeScript RT reagent Kit (TaKaRa, Dalian, China). Real-time quantitative PCR analyses were carried out using SYBR Premix Ex Taq II (TaKaRa). Bulge-loop^TM^ miRNA qRT-PCR Primer Sets (one RT primer and a pair of qPCR primers for each set) specific for miR-3662 and U6 were designed by RiboBio (Guangzhou, China). The relative expression levels of miRNA and mRNA expression were normalized to U6 and β-actin, respectively.

### Establishment of stably transfected cells

The miR-3662-NC, pre-miR-3662, miR-3662-inhibitor, LV-HIF-1α, and LV-NC lentivirus vectors were purchased from GenePharma (Shanghai, China). The miR-3662-NC included a non-targeting sequence. The nature of the inhibitor was short hairpin RNA. Lentiviruses were infected into HCC cells with a multiplicity of infection ranging from 10 to 20 in the presence of polybrene (10 μg/mL, GenePharma). At 72 h after infection, cells were selected for seven days using puromycin (5 μg/mL, Sigma-Aldrich).

### Lactate production, glucose uptake, cellular glucose-6-phosphate (G6P), and ATP levels

Cells were cultured in DMEM without phenol red for 15 h, and the culture media was harvested for measurement of lactate or glucose concentrations. Lactate levels were measured using the Lactate Assay kit (BioVision, Mountain View, CA, USA), and glucose levels were quantified with a glucose assay kit (Sigma-Aldrich). The levels of cellular G6P were measured using the Glucose-6-phosphate Fluorometric Assay kit (Cayman, Ann Arbor, MI, USA), and ATP levels were determined with CellTiter-Glo Luminescent Cell Viability Assay (Promega, Madison, MI, USA). Independent experiments were repeated three times.

### Extracellular acidification and oxygen consumption rate assays

The extracellular acidification rate (ECAR) and cellular oxygen consumption rate (OCR) were measured using the Seahorse XF 96 Extracellular Flux Analyzer (Agilent Technologies, Santa Clara, CA, USA) according to the manufacturer’s instructions. Seahorse XF Glycolysis Stress Test Kit and Seahorse XF Cell Mito Stress Test Kit were used to determine ECAR and OCR, respectively. Briefly, 1 × 10^4^ cells/well were seeded into a Seahorse XF 96 cell culture microplate, allowed to adhere overnight. Before the assay, the cells were washed with assay medium (unbuffered DMEM supplemented with 2 mM L-glutamine, pH = 7.4) and incubated in a CO_2_-free incubator at 37 °C for 1 h. Then the microplates were loaded into the Seahorse Analyzer. The measurement cycles were 3, and each measurement cycle consisted of 4.5 min Mix, 0 min Wait, and 4.5 min Measure. For ECAR, glucose (10 mM), the oxidative phosphorylation inhibitor oligomycin (1.0 μM), and the glycolytic inhibitor 2-deoxyglucose (2-DG, 50 mM) were sequentially injected into each well at indicated time points. For OCR, oligomycin (1.0 μM), the mitochondrial uncoupler carbonyl cyanide *p*-trifluoromethoxy phenylhydrazone (FCCP, 1.0 μM), and the mitochondrial complex I inhibitor rotenone plus the mitochondrial complex III inhibitor antimycin A (Rote/AA, 0.5 μM) were sequentially injected. After the assays, the number of cells was calculated using the crystal violet assay. All measurements were normalized to cell number. Glycolysis rate = (Maximum rate measurement before oligomycin injection)−(Last rate measurement before glucose injection). Glycolytic capacity =  (Maximum rate measurement after oligomycin injection)−(Last rate measurement before glucose injection). Basal respiration = (Last rate measurement before first injection)−(Minimum rate measurement after Rote/AA injection). Maximal respiration = (Maximum rate measurement after FCCP injection)−(Minimum rate measurement after Rote/AA injection). ECAR is shown in mpH/min and OCR in pMoles/min. Three replicates were performed for each group, and independent experiments were repeated three times.

### Cell Counting Kit-8 (CCK-8) assay

Cell growth was evaluated by a CCK-8 Kit (Dojindo Laboratories, Kumamoto, Japan) according to the manufacturer’s protocols. Briefly, cells were seeded in 96-well plates at a density of 1 × 10^3^ cells per well with 100 μl culture medium. After treatment with the hypoxia mimetic CoCl_2_, 10 μl of CCK-8 solution was added to each well at indicated time points (1, 2, 3, 4, and 5 d). The plates were incubated at 37 °C for 2 h, and absorbance at 450 nm was measured. Three duplicates were performed for each group, and independent experiments were repeated three times.

### 5-ethynyl-2′-deoxyuridine (EdU) incorporation assay

After CoCl_2_ treatment, cells (2 × 10^5^) were incubated with EdU (RiboBio) for 2 h at 37 °C. Then the cells were fixed in 4% paraformaldehyde. After permeabilization with 0.5% Triton-X, the cells were reacted with 1 × Apollo reaction cocktail (RiboBio) for 30 min. Subsequently, the DNA contents of the cells were stained with Hoechst 33342 for 30 min and visualized under a fluorescence microscope. EdU positive cells were counted from three random microscopic fields for each well, and these experiments were repeated three times independently.

### Colony formation assay

Cells (500 cells/well) were treated with 150 μM CoCl_2_, and seeded in each well of a six-well cell culture plate. After 2 weeks, the plates were fixed in 4% paraformaldehyde and stained with 1% crystal violet. The colony numbers were counted to assess cell proliferation. The assays were performed in three independent experiments.

### Flow cytometry analysis

After CoCl_2_ treatment, cells were fixed in 75% cold ethanol after incubation, and were stored at 4 °C overnight. The fixed cells were washed with phosphate-buffered saline twice, and stained with propidium iodide for 30 min. Cell cycle distribution was analyzed by flow cytometry (FACSCalibur, Becton Dickinson, San Jose, CA, USA). The data of cell cycle was analyzed using Kaluza Flow Cytometry Analysis Software version 1.2 (Beckman Coulter, Brea, CA, USA). Three independent experiments were performed.

### Western blotting

Cells were treated with 150 mM CoCl_2_, and proteins were extracted using radioimmunoprecipitation assay buffer containing protease and phosphatase inhibitor cocktails. Proteins were separated on a sodium dodecyl sulfate-polyacrylamide gel and transferred onto a polyvinylidene difluoride membrane (Bio-Rad, Hercules, CA, USA). Then the membrane was blocked with 5% non-fat milk, and incubated with primary antibodies at 4 °C overnight. Antibodies against *p*-ERK (#4370, Cell Signaling Technology, Beverly, MA, USA), ERK (#4695, Cell Signaling Technology), *p*-JNK (#4668, Cell Signaling Technology), JNK (#9252, Cell Signaling Technology), HIF-1α (ab2185, Abcam, Cambridge, UK), GLUT1 (ab115730, Abcam), HK2 (#2867, Cell Signaling Technology), PKM2 (#4053, Cell Signaling Technology), and LDHA (#3582, Cell Signaling Technology) were used, and β-tubulin (ab6046, Abcam) served as an internal control for immunoblot. The membrane was incubated with the appropriate horseradish peroxidase-conjugated secondary antibodies the next day. The proteins were detected using enhanced chemiluminescence detection kit (EMD Millipore, Billarica, MA, USA).

### Immunohistochemistry

Tissue sections were deparaffinized, rehydrated, and treated with 3% H_2_O_2_ for 15 min to inhibit endogenous peroxidase activity. Following heat-induced epitope retrieval in 10 mM citrate buffer (pH 6.0) in a microwave for 30 min, the slides were incubated at 4 °C overnight with a prediluted primary antibody (HIF-1α, ab114977, Abcam; Ki-67, #9027, Cell Signaling Technology). After incubation with a secondary antibody, the signal was developed with 3,3′-diaminobenzidine tetrachloride.

### Luciferase reporter assay

The 3′-untranslated region (3′-UTR) of human HIF-1α containing putative binding sites (UGCAUUGCAGUAGCAUCAUUUUA) was cloned into the pGL3 plasmid (Ambion, Austin, TX, USA), with the resulting expression vectors being named WT-HIF-1α-3′-UTR. An altered HIF-1α 3′-UTR carrying a mutation in the miR-3662 binding sequence (UGCAUUGCAGUAGCACACGCGGA) was created and inserted into the pGL3 vector, with the resulting construct being named MUT-HIF-1α-3′-UTR. Cells were seeded in 24-well plates (5 × 10^5^ cells per well) and incubated for 24 h before transfection. Cells were co-transfected with 120 ng of either pGL3-HIF-1α-WT or pGL3-HIF-1α-MUT reporter plasmids together with 40 nM of miR-3662 mimic or negative control oligoribonucleotides using Lipofectamine 3000 (Invitrogen). Cells were also transfected with 10 ng of pRL-TK plasmid (Promega) for internal normalization. Cells were collected after 48 h and lysed using the lysis buffer (Promega). The luciferase reporter assay was conducted using the Dual-Luciferase Reporter Assay System (Promega), in accordance with the manufacturer’s instructions.

### Xenograft in nude mice

4-week-old BALB/c nude mice (*n* = 12) were purchased from Animal Core Facility of Nanjing Medical University (Nanjing, China). Suspended in 100 μL serum-free DMEM, 5 × 10^6^ tumor cells were subcutaneously injected into the flanks of the mice. All mice were monitored once every 3 days and were sacrificed 4 weeks later. Tumor volume was measured as follows: volume = length × width^2^ × 0.5. All animal studies were approved by the Institutional Animal Care and Use Committee of Nanjing Medical University.

### Statistical analysis

Data are presented as the mean ± standard error of the mean (S.E.M.) from at least three independent experiments. Differences between groups were analyzed using Student *t* test. Paired *t* test was used to analyze miR-3662 and HIF-1α mRNA levels in tissue samples. Spearman correlation test was preformed to analyze the correlation between miR-3662 and HIF-1α mRNA levels. All statistical analyses were performed using SPSS 21.0 (IBM SPSS software, NY, USA) and Prism 6 (GraphPad Software, La Jolla, CA, USA). Statistically significance was defined as *P* < 0.05 (*), *P* < 0.01 (**), and *P* < 0.001 (***).

## Results

### miR-3662 expression is downregulated in HCC tissues and cell lines

**Fig. 1:**
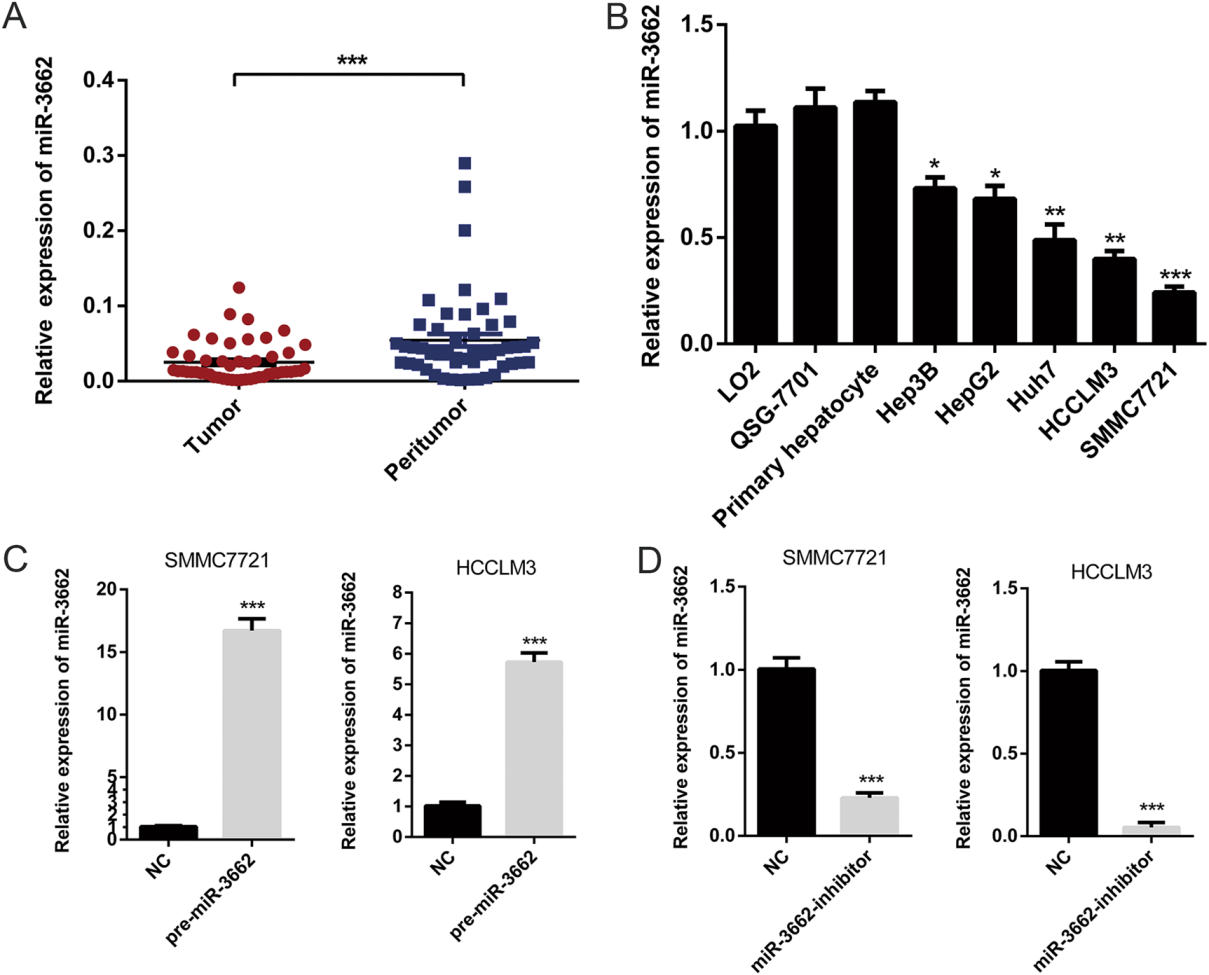
miR-3662 expression level is decreased in HCC tissue samples and cell lines. **a** RT-qPCR was used to detect the expression of miR-3662 in 50 pairs of HCC tissues and corresponding peritumor tissues. ***P < 0.001 compared with the corresponding peritumor tissues. **b** The expression levels of miR-3662 in five HCC cell lines (Hep3B, HepG2, Huh7, HCCLM3, and SMMC7721), two human liver cell lines (LO2 and QSG-7701), and primary human hepatocytes. Three independent experiments were performed per group. *P < 0.05, **P < 0.01, ***P < 0.001 compared with the expression level of miR-3662 in LO2 cells. **c**, **d** SMMC7721 and HCCLM3 cells were transfected with lentivirus overexpressing miR-3662 (defined as pre-miR-3662) (**c**) or lentivirus with short hairpin RNA targeting miR-3662 (defined as miR-3662-inhibitor) (**d**), respectively. The negative control (NC) cells included a non-targeting sequence. miR-3662 expression levels were analyzed by RT-qPCR from three independent experiments. ***P < 0.001 compared with the miR-3662 expression level in the NC group. All data are represented as the means ± S.E.M

miR-3662 has been reported to function as a tumor suppressor and an oncogene in different malignancies^11–13^, but very little is known about the role of miR-3662 in HCC. To investigate the clinicopathological significance of miR-3662 in HCC, we first examined the expression patterns of miR-3662 in 50 pairs of HCC tissues and matched adjacent nontumorous tissues by RT-qPCR. As shown in Fig. 1a, miR-3662 expression level was significantly downregulated in HCC samples compared to that in peritumor samples. All patients were divided into high expression group and low expression group using the median level of miR-3662 as the cutoff value. Investigation of the correlation between the expression of miR-3662 and clinicopathological features showed that low levels of miR-3662 were significantly associated with large tumor size (*P* *=* 0.022), tumor multiplicity (*P* *=* 0.019), advanced Edmondson grade (*P* *=* 0.032), and high tumor-node-metastasis stage (*P* *=* 0.004) (Table 1). Compared with the miR-3662 expression in normal liver cell lines (LO2 and QSG-7701) and primary hepatocytes, the expression levels of miR-3662 were significantly lower in HCC cell lines, including Hep3B, HepG2, Huh7, HCCLM3, and SMMC7721 (Fig. 1b). Notably, HCCLM3 and SMMC7721 possessed much lower miR-3662 levels. Therefore, we used HCCLM3 and SMMC7721 cells as models to investigate the effect of miR-3662 on HCC progression.

**Table 1:** Association of miR-3662 expression with various clinical parameters in HCC patients

Clinicopathological features	*n*	Low miR-3662	High miR-3662	*P*
Age (years)				0.567
≤60	21	12 (57.1%)	9 (42.9%)	
>60	29	13 (44.8%)	16 (55.2%)	
Gender				1.000
Female	15	7 (46.7%)	8 (53.3%)	
Male	35	18 (51.4%)	17 (48.6%)	
Liver cirrhosis				0.667
No	6	4 (66.7%)	2 (33.3%)	
Yes	44	21 (47.7%)	23 (52.3%)	
HBsAg status				1.000
Negative	7	3 (42.9%)	4 (57.1%)	
Positive	43	22 (51.2%)	21 (48.8%)	
α-fetoprotein (ng/ml)				0.345
≤20	14	5 (35.7%)	9 (64.3%)	
>20	36	20 (55.6%)	16 (44.4%)	
Tumor size (cm)				0.022
≤5	23	7 (30.4%)	16 (69.6%)	
>5	27	18 (66.7%)	9 (33.3%)	
Tumor multiplicity				0.019
Single	31	11 (35.5%)	20 (64.5%)	
Multiple	19	14 (73.7%)	5 (26.3%)	
Edmondson grade				0.032
I–II	34	13 (38.2%)	21 (61.8%)	
III–IV	16	12 (75.0%)	4 (25.0%)	
Tumor-node-metastasis stage				0.004
I–II	29	9 (31.0%)	20 (69.0%)	
III	21	16 (76.2%)	5 (23.8%)	

Taken together, these data suggested that the expression levels of miR-3662 were downregulated in HCC tissues and cell lines, and miR-3662 expression level was significantly associated with clinicopathological features.

### miR-3662 suppresses the Warburg effect in HCC

To further examine the impact of miR-3662 on the malignant phenotypes of liver cancer, we constructed both miR-3662 overexpression and knockdown cell lines using lentiviral-based approaches. Overexpression or knockdown efficiency was confirmed by RT-qPCR. As indicated in Fig. 1c, d, the expression of miR-3662 was upregulated ~17-fold in SMMC7721 cells, and decreased to nearly 5% in HCCLM3 cells.

**Fig. 2:**
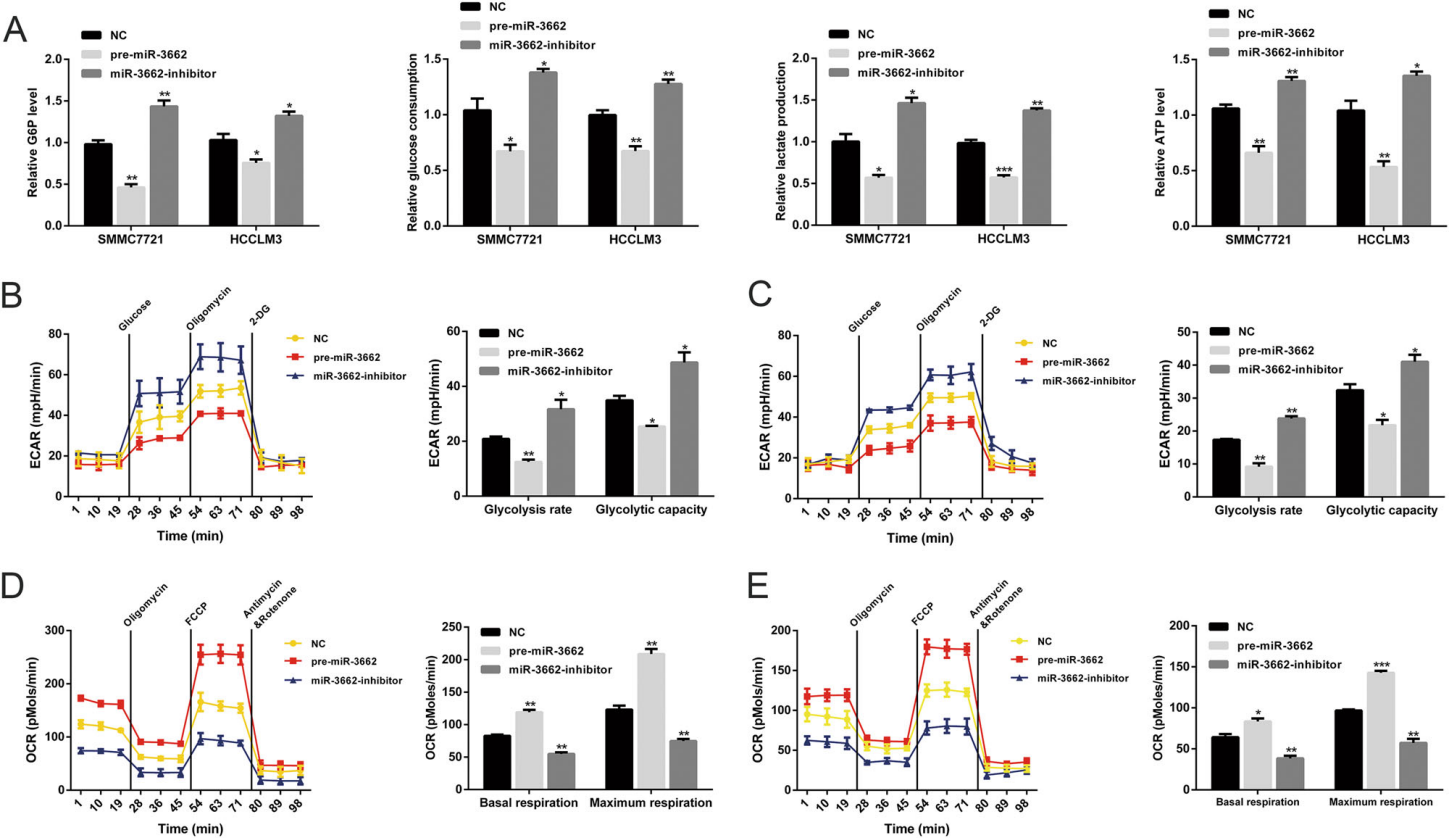
miR-3662 inhibits the Warburg effect in HCC. **a** Cellular G6P level, glucose consumption, lactate production, and cellular ATP level in SMMC7721 and HCCLM3 cells with miR-3662 overexpression or miR-3662 knockdown. Three independent experiments were performed. *P < 0.05, **P < 0.01, ***P < 0.001 compared with the negative control. **b**, **c** The ECAR data showing the glycolysis rate and glycolytic capacity in miR-3662 overexpressing or silencing SMMC7721 (**b**) and HCCLM3 (**c**) cells (three replicates for each group, and three independent experiments were performed). Glucose (10 mM), the oxidative phosphorylation inhibitor oligomycin (1.0 μM), and the glycolytic inhibitor 2-deoxyglucose (2-DG, 50 mM) were sequentially injected into each well at indicated time points. All measurements were normalized to cell number calculated using the crystal violet assay at the end of the experiment. *P < 0.05, **P < 0.01 compared with the negative control. **d**, **e** The OCR results showing the basal respiration and maximum respiration in miR-3662 overexpressing or silencing SMMC7721 (**d**) and HCCLM3 (**e**) cells (three replicates for each group, and three independent experiments were performed). Oligomycin (1.0 μM), the mitochondrial uncoupler carbonyl cyanide *p*-trifluoromethoxy phenylhydrazone (FCCP, 1.0 μM), and the mitochondrial complex I inhibitor rotenone plus the mitochondrial complex III inhibitor antimycin A (Rote/AA, 0.5 μM) were sequentially injected. After the assays, the number of cells was calculated using the crystal violet assay. All measurements were normalized to cell number at the end of the experiment. **P < 0.01, ***P < 0.001 compared with the negative control. All data are presented as the means ± S.E.M

Given that the Warburg effect is a well-characterized metabolic shift that ubiquitously occurs in tumor cells, we next explored the role of miR-3662 in HCC cell glucose metabolism. As shown in Fig. 2a, miR-3662 overexpression dramatically reduced the cellular G6P level, glucose consumption, lactate production, and cellular ATP level in liver cancer cells, whereas knockdown of miR-3662 led to the opposite results in SMMC7721 and HCCLM3 cells.

To further validate the impact of miR-3662 on HCC glycolysis, ECAR was measured using the Seahorse XF 96 Extracellular Flux Analyzer. Glycolysis rate and glycolytic capacity can be ascertained by the ECAR data. Overexpression of miR-3662 significantly reduced glycolysis rate and glycolytic capacity of SMMC7721 and HCCLM3 cells. Conversely, silencing miR-3662 remarkably elevated both (Fig. 2b, c). In addition, we examined OCR, an indicator of mitochondrial respiration. The results showed that pre-miR-3662-transfected cells displayed increased OCR, whereas knockdown of miR-3662 suppressed OCR in SMMC7721 and HCCLM3 cells (Fig. 2d, e).

### miR-3662 affects HCC cell proliferation and cell cycle in vitro

**Fig. 3:**
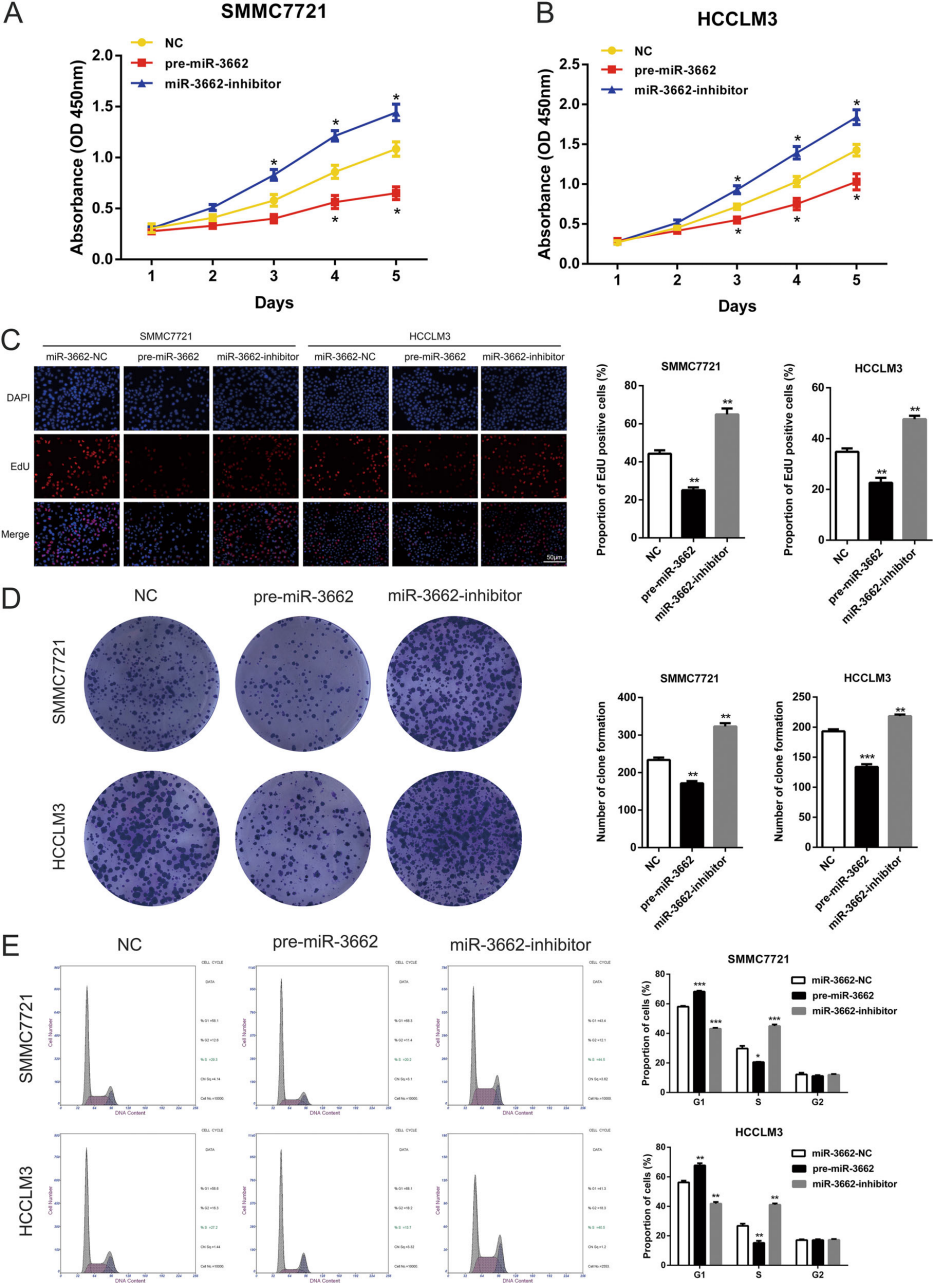
miR-3662 suppresses HCC cell proliferation and induces cell cycle arrest. **a**, **b** CCK-8 assays in SMMC7721 (**a**) and HCCLM3 (**b**) cells overexpressing or silencing miR-3662. Three duplicates were performed for each group, and three independent experiments were performed. *P < 0.05 compared with the negative control. **c** 5-ethynyl-2′-deoxyuridine (EdU) incorporation assays were performed to assess the cell proliferation in miR-3662 overexpressing or silencing SMMC7721 and HCCLM3 cells. EdU positive cells were counted from three random microscopic fields for each well, and these experiments were repeated three times independently. **P < 0.01 compared with the negative control. **d** Colony formation ability of miR-3662 overexpressing or silencing SMMC7721 and HCCLM3 cells. Three independent experiments were performed for each group. **P < 0.01, ***P < 0.001 compared with the negative control. **e** Flow cytometry results showing the cell cycle distribution of miR-3662 overexpressing or silencing SMMC7721 and HCCLM3 cells. Three independent experiments were performed for each group. *P < 0.05, **P < 0.01, ***P < 0.001 compared with the negative control. All data are represented as the means ± S.E.M

CCK-8 assays showed that miR-3662 overexpression remarkably inhibited cell proliferation in SMMC7721 and HCCLM3, whereas knockdown of miR-3662 significantly enhanced the growth of HCC cells (Fig. 3a, b). Concordant with these results, EdU assays showed impaired growth of HCC cells with ectopic miR-3662 expression. Conversely, silencing miR-3662 increased cell proliferation in SMMC7721 and HCCLM3 cells (Fig. 3c). In addition, the colony formation assays revealed compromised colony-forming ability of liver cancer cells transfected with pre-miR-3662 lentivirus, whereas miR-3662 knockdown promoted cell growth compared with control cells (Fig. 3d).

Because miR-3662 markedly attenuated HCC cell proliferation, we next aimed to determine whether miR-3662 affected cell cycle progression of HCC cells. We quantified cell cycle distribution using flow cytometry, and found that overexpression of miR-3662 induced G1-phase arrest in both SMMC7721 and HCCLM3 cells. In consistency with the aforementioned data, it was observed that miR-3662 knockdown led to a remarkable decrease in the cellular population of G1 phase with a significant increase in S phase in liver cancer cells (Fig. 3e).

### miR-3662 inhibits the ERK/JNK signaling pathway

**Fig. 4:**
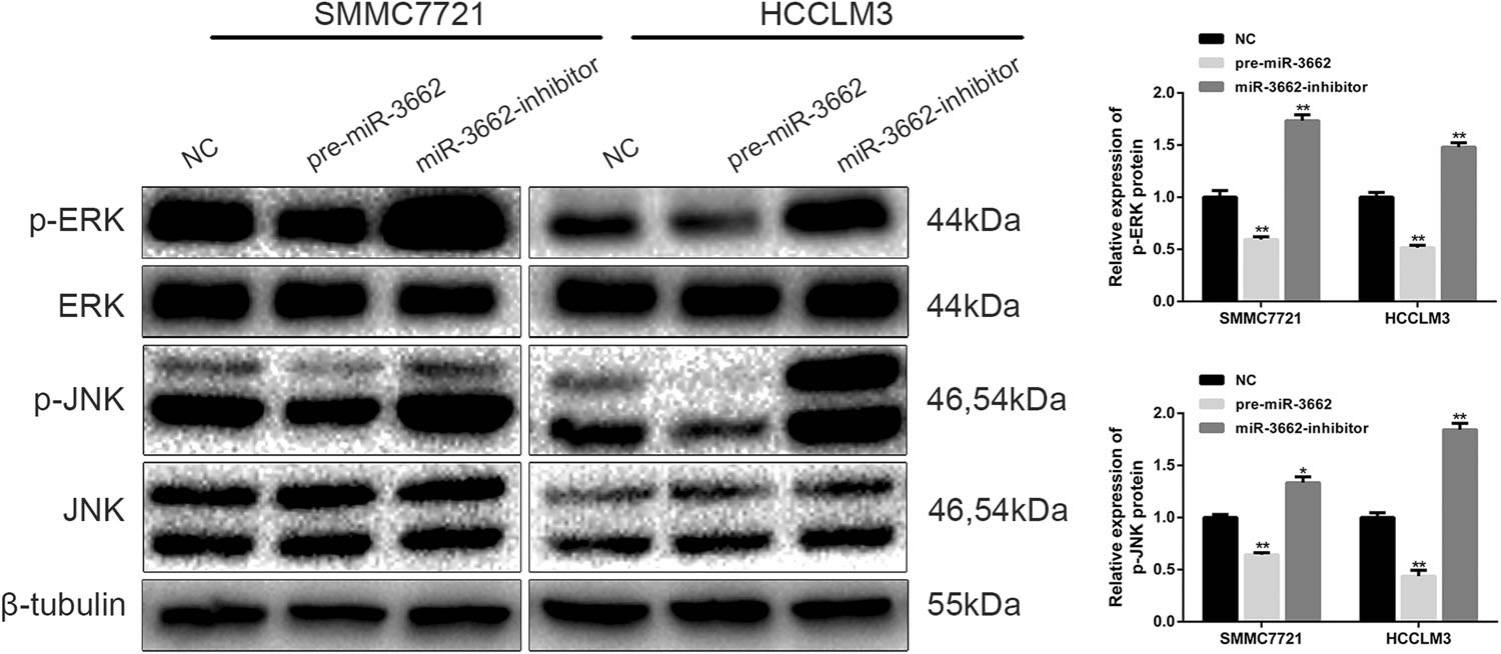
miR-3662 inhibits the activation of the ERK and JNK signaling pathways. Left panel: Representative western blotting bands showing the expression levels of *p*-ERK, ERK, *p*-JNK, JNK, and β-tubulin in SMMC7721 and HCCLM3 cells with upregulated or downregulated miR-3662 expression. Three independent experiments were performed. Right panel: the protein bands were quantified according to the results of three independent western blotting experiments. The ratio of *p*-ERK to ERK, and the ratio of *p*-JNK to JNK were then calculated. The relative protein expression levels are shown as histograms. *P < 0.05, **P < 0.01 compared with the negative control group. All data are represented as the means ± S.E.M

The ERK and JNK signaling pathways, frequently dysregulated in tumorigenesis, are crucial in the regulation of cell proliferation. To further explore the potential signaling pathways involved in miR-3662-associated HCC progression, we investigated the influence of miR-3662 overexpression and knockdown on the ERK/JNK signaling pathway using western blotting. As shown in Fig. 4, the expression levels of *p*-ERK and *p*-JNK were decreased in SMMC7721-pre-miR-3662 and HCCLM3-pre-miR-3662 cells, whereas the protein levels of *p*-ERK and *p*-JNK were significantly increased in SMMC7721-miR-3662-inhibitor and HCCLM3-miR-3662-inhibitor cells. Taken together, the data suggested that miR-3662 regulated the ERK/JNK signaling pathway in liver cancer.

### HIF-1α is a direct target of miR-3662

**Fig. 5:**
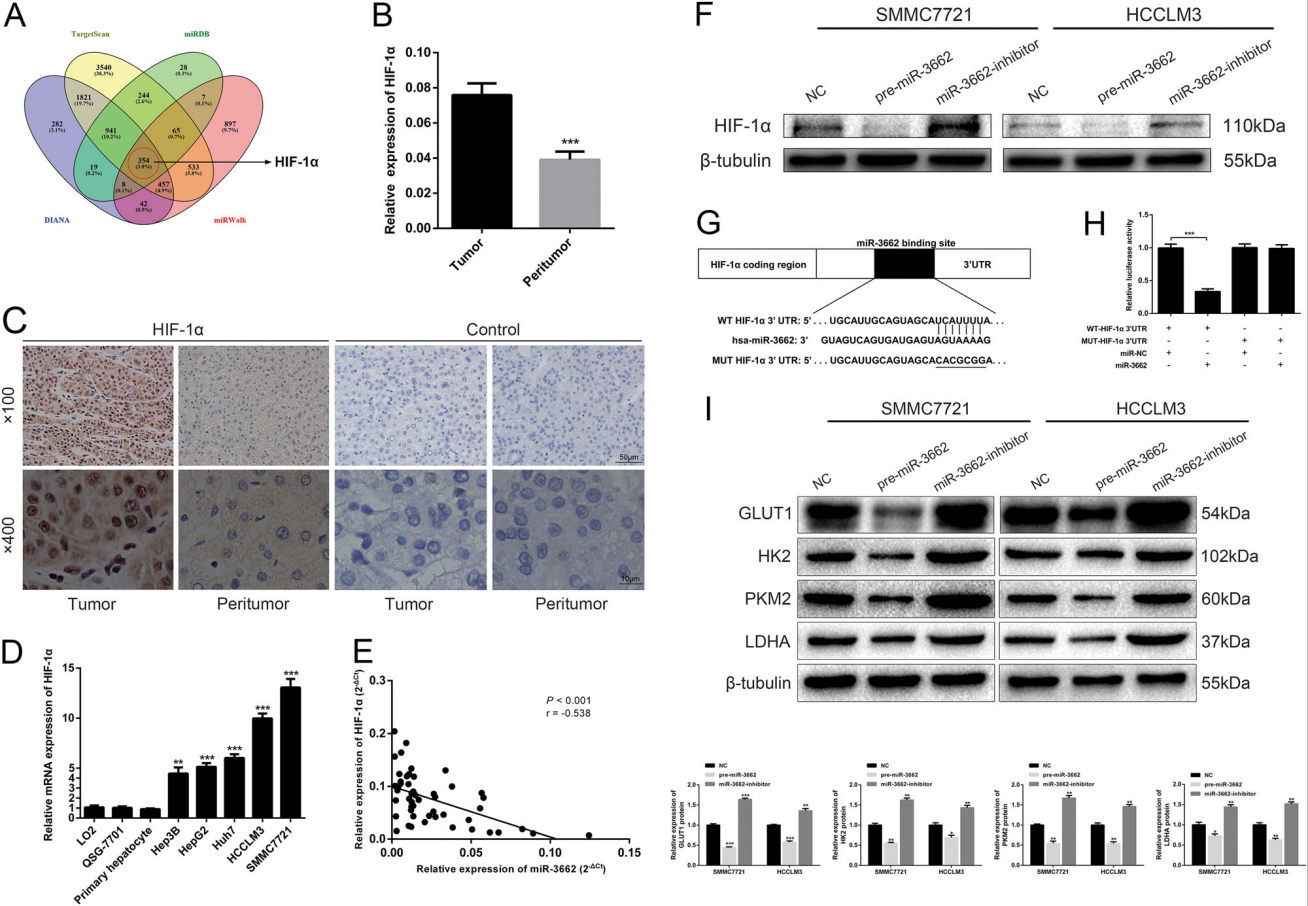
miR-3662 directly targets HIF-1α in HCC cells. **a** Venn diagram displaying miR-3662 computationally predicted to target HIF-1α by four different prediction algorithms: TargetScan, miRDB, DIANA, and miRWalk. **b** The expression levels of HIF-1α mRNA in 50 pairs of HCC tissues and corresponding peritumor tissues were measured by RT-qPCR. ***P < 0.001 compared with the corresponding peritumor tissues. **c** Representative immunostaining images of HIF-1α in HCC tissues and the corresponding peritumor samples. Immunostaining images using secondary antibody alone are shown as the control. **d** HIF-1α mRNA expression levels were examined in five HCC cell lines (Hep3B, HepG2, Huh7, HCCLM3, and SMMC7721), two human liver cell lines (LO2 and QSG-7701), and primary human hepatocytes. Three independent experiments were performed per group. **P < 0.01, ***P < 0.001 compared with the expression level of HIF-1α mRNA in LO2 cells. **e** Spearman correlation analysis was employed to confirm the correlations between the HIF-1α mRNA and miR-3662 expression levels in 50 HCC samples (*r* = −0.538, *P* < 0.001). **f** Western blotting analysis indicated that HIF-1α expression levels were decreased in SMMC7721 and HCCLM3 cells with miR-3662 overexpression, but increased in miR-3662-silenced SMMC7721 and HCCLM3 cells. β-tubulin was used as the loading control. **g** Predicted miR-3662 targeting sequence in HIF-1α 3′-UTR (WT HIF-1α 3′-UTR). Target sequences of HIF-1α 3′-UTR were mutated (MUT HIF-1α 3′-UTR). **h** Dual-luciferase reporter assay of cells transfected with WT HIF-1α 3′-UTR or MUT HIF-1α 3′-UTR reported together with 40 nM of miR-3662 mimic or negative control oligoribonucleotides (three independent experiments per group). ***P < 0.001 compared with the relative luciferase activity in WT HIF-1α 3′-UTR plus miR-NC group. **i** Western blotting analysis of the expression levels of GLUT1, HK2, PKM2, and LDHA in miR-3662 overexpressed, miR-3662 silenced and control HCC cells. Qualitative changes from three independent experiments are shown as histograms. *P < 0.05, **P < 0.01, ***P < 0.001 compared with cells transfected with negative control. All data are represented as the means ± S.E.M

Next, we explored the molecular mechanisms responsible for the functions of miR-3662 that were observed above. TargetScan, miRDB, DIANA, and miRWalk were employed to identify potential miR-3662 target genes (Fig. 5a and Supplementary Table 1). Among the potential target genes, HIF-1α was predicted by all four databases. HIF-1α was of particular interest because it plays a crucial role in the reprogramming of cancer metabolism^16^. As HIF-1α was previously reported to be target of several miRNAs in HCC^17–19^, we postulated that miR-3662 may exert its anti-tumor function through directly targeting HIF-1α.

We analyzed HIF-1α expression in 50 pairs of HCC tissues and corresponding peritumor tissues using RT-qPCR, and found that the expression level of HIF-1α was markedly higher in HCC tissues (Fig. 5b). Higher HIF-1α expression in HCC tissues was further confirmed using immunohistochemistry (Fig. 5c), which was in consistence with previously reported data^17^. HIF-1α mRNA expression levels were also examined using five HCC cell lines and two human liver cell lines as well as primary human hepatocytes. As indicated in Fig. 5d, HIF-1α mRNA expression level was higher in HCC cell lines compared to that in normal liver cell lines and primary hepatocytes. In addition, an inverse correlation between miR-3662 and HIF-1α mRNA was observed in HCC tissues (Fig. 5e).

To verify HIF-1α was a bona fide downstream target of miR-3662, we investigated the influence of altered miR-3662 level on the expression of HIF-1α. It was revealed that miR-3662 overexpression resulted in decreased HIF-1α protein level, while miR-3662 knockdown led to the opposite results after treatment with the hypoxia mimetic CoCl_2_ (Fig. 5f). Then we carried out a luciferase reporter assay to corroborate the direct interaction between miR-3662 and HIF-1α. WT or MUT 3′-UTR target sequences were cloned into a luciferase reporter vector. Results showed that miR-3662 inhibited the luciferase activity of the WT 3′-UTR of HIF-1α (Fig. 5g, h).

Moreover, we investigated whether miR-3662 affected the expression of HIF-1α downstream effectors involved in glycolytic regulation. As examined by western blotting, we found that miR-3662 overexpression reduced the expression levels of GLUT1, HK2, PKM2, and LDHA after treatment with the hypoxia mimetic CoCl_2_. Consistently, suppression of miR-3662 elevated the expression levels of GLUT1, HK2, PKM2, and LDHA in liver cancer cells (Fig. 5i).

### HIF-1α ameliorates the inhibitory effect of miR-3662 on HCC progression

**Fig. 6:**
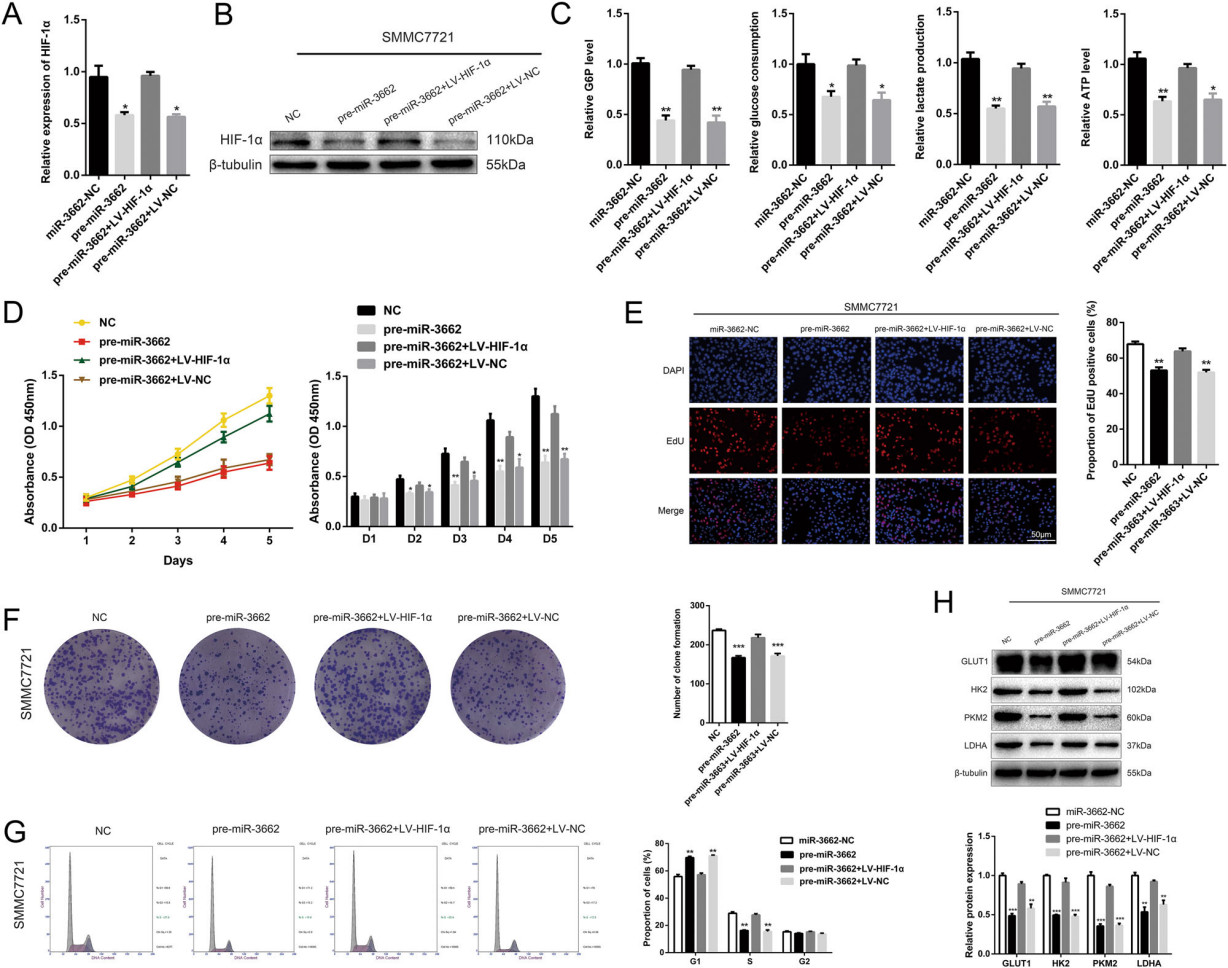
Rescue experiments are performed to confirm that HIF-1α is the functional target of miR-3662 on HCC progression. **a**, **b** Expression of HIF-1α at the mRNA and protein in the SMMC7721 cells transfected with miR-3662 overexpression lentivirus (pre-miR-3662) and HIF-1α overexpression lentivirus (LV-HIF-1α). Experiments were performed in triplicate. **P < 0.01 compared with the negative control group. **c** Cellular G6P level, glucose consumption, lactate production, and cellular ATP level in SMMC7721 cells co-transfected with pre-miR-3662 lentivirus and LV-HIF-1α (three independent experiments per group). *P < 0.05, **P < 0.01 compared with the negative control group. **d** CCK-8 analysis of SMMC7721-pre-miR-3662 cells transfected with LV-HIF-1α or LV-NC. Three duplicates were performed for each group, and three independent experiments were performed. *P < 0.05, **P < 0.01 compared with the negative control group. **e** 5-ethynyl-2′-deoxyuridine (EdU) incorporation analysis of miR-NC, pre-miR-3662, pre-miR-3662 plus LV-HIF-1α, and pre-miR-3662 plus LV-NC in SMMC7721 cells. EdU positive cells were counted from three random microscopic fields for each well, and these experiments were repeated three times independently. **P < 0.01 compared with the negative control group. **f** Colony formation ability of SMMC7721-pre-miR-3662 cells transfected with LV-HIF-1α or LV-NC (three independent experiments per group). ***P < 0.001 compared with the negative control group. **g** Cell cycle analysis of SMMC7721-pre-miR-3662 cells transfected with LV-HIF-1α or LV-NC. Three experiments were performed for each group. Three independent experiments were performed. **P < 0.01 compared with the negative control group. **h** Western blotting analysis of GLUT1, HK2, PKM2, and LDHA protein levels in SMMC7721-pre-miR-3662 cells transfected with LV-HIF-1α or LV-NC. Three independent experiments were performed. **P < 0.01, ***P < 0.001 compared with the negative control group. All data are represented as the means ± S.E.M

To further elucidate that miR-3662 regulated HCC progression by targeting HIF-1α, we transfected SMMC7721-pre-miR-3662 cells with LV-HIF-1α, and HIF-1α overexpression was verified by RT-qPCR and western blotting (Fig. 6a, b). We found that ectopic HIF-1α expression rescued miR-3662-mediated reduction in cellular G6P level, glucose consumption, lactate production, and cellular ATP level in SMMC7721 cells (Fig. 6c). Importantly, reintroduction of HIF-1α significantly abolished the inhibitory effects of miR-3662 on cell proliferation according to the results from CCK-8 assays, EdU assays, and colony formation assays (Fig. 6d–f). Moreover, HIF-1α overexpression reversed G1/S cell cycle arrest in SMMC7721 cells (Fig. 6g). Suppressed expression levels of GLUT1, HK2, PKM2, and LDHA were restored by upregulation of HIF-1α after treatment with the hypoxia mimetic CoCl_2_ (Fig. 6h). Collectively, our results supported the findings that the miR-3662/ HIF-1α axis played a key role in regulating Warburg effect, proliferation process, and cell cycle distribution in liver cancer cells.

### miR-3662 suppresses xenograft tumor growth in vivo

**Fig. 7:**
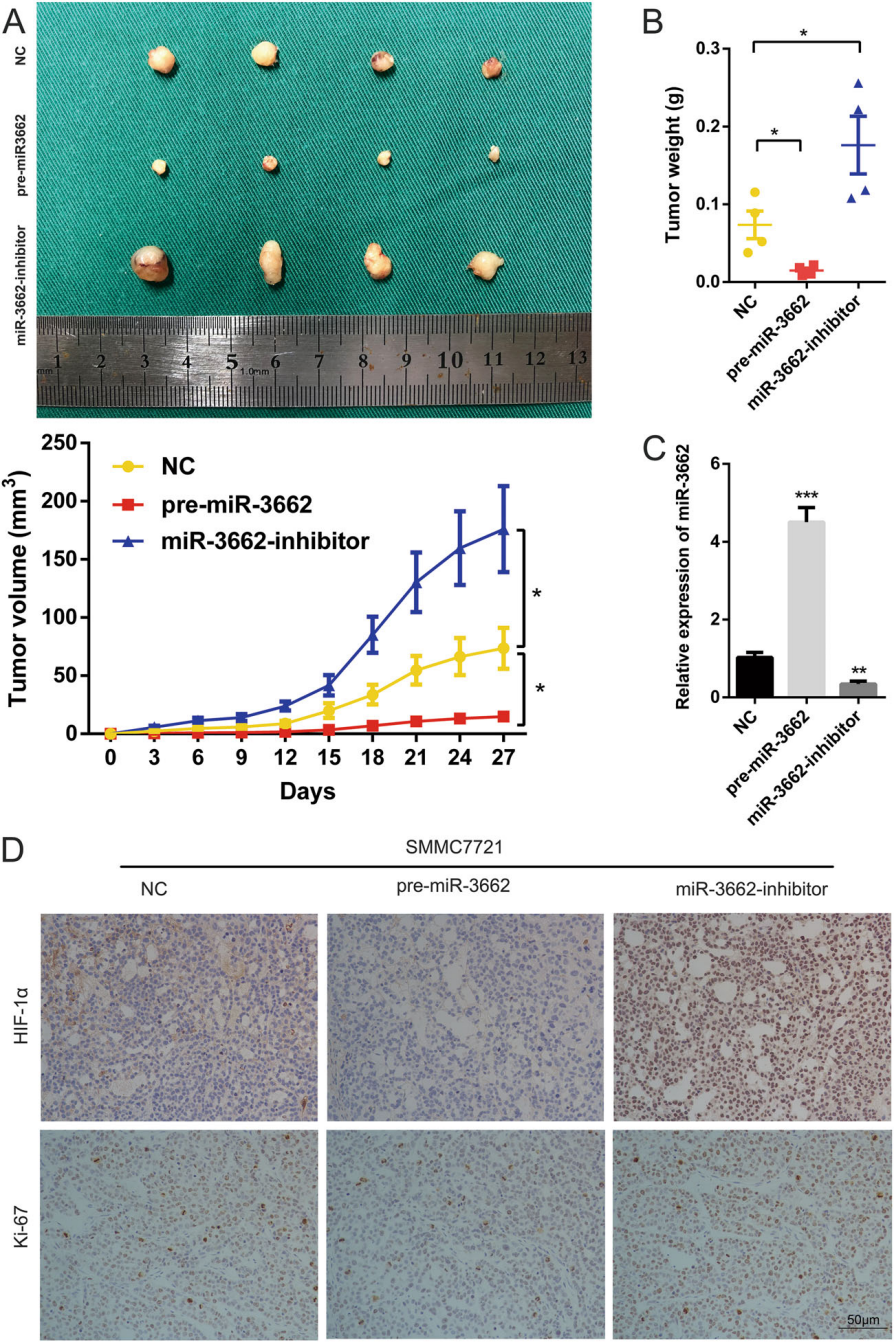
miR-3662 suppresses xenograft tumor growth in vivo. **a** Upper panel: photographs of tumors derived from mice inoculated with SMMC7721-miR-3662-NC, SMMC7721-pre-miR-3662, and SMMC7721-miR-3662 inhibitor cells (*n* = 4 per group). Lower panel: growth curves for tumor volumes are shown (*n* = 4 per group). Tumor volume was calculated based on the following equation: Volume (mm^3^) = Length (mm) × Width^2^ (mm^2^) × 0.5. *P < 0.05 compared with the SMMC7721-miR-3662-NC group. **b** Tumor weight of the xenografts derived from mice inoculated with SMMC7721-miR-3662-NC, SMMC7721-pre-miR-3662, and SMMC7721-miR-3662 inhibitor cells (*n* = 4 per group). *P < 0.05 compared with the SMMC7721-miR-3662-NC group. **c** miR-3662 expression levels were analyzed by RT-qPCR in SMMC7721-miR-3662-NC, SMMC7721-pre-miR-3662, and SMMC7721-miR-3662 inhibitor xenografts. Three independent experiments were performed for each group. *P < 0.05, ***P < 0.001 compared with the SMMC7721-miR-3662-NC group. **d** Representative immunostaining images measuring HIF-1α and Ki-67 levels in tumors derived from mice inoculated with SMMC7721-miR-3662-NC, SMMC7721-pre-miR-3662, and SMMC7721-miR-3662 inhibitor cells. All data are presented as the means ± S.E.M

To examine the growth of tumor cells in vivo, we constructed xenograft animal model by implanting the SMMC7721 cells with stable overexpression or knockdown of miR-3662 into nude mice. Each mouse was monitored once every three days, and the mice were euthanized after 4 weeks. Compared with tumors derived from SMMC7721-miR-3662-NC cells, those derived from SMMC7721-pre-miR-3662 cells grew slower. Tumor weight was significantly lower in mice inoculated with SMMC7721-pre-miR-3662 cells at the fourth week compared with that in control group (Fig. 7a, b). Conversely, in mice inoculated with SMMC7721-miR-3662-inhibitor cells, average tumor volume was significantly larger at the fourth week than that in control mice, and tumors were also heavier (Fig. 7a, b).

Furthermore, we detected the expression of miR-3662 in xenografts using RT-qPCR. As shown in Fig. 7c, miR-3662 expression was increased in the pre-miR-3662-treated group, whereas lower miR-3662 level was observed in miR-3662-inhibitor-treated group. Xenografts were also subjected to immunohistochemistry. Results showed that HIF-1α expression level was decreased in the pre-miR-3662-treated group, whereas elevated expression level of HIF-1α was detected in miR-3662-inhibitor-treated group (Fig. 7d). Ki-67 staining of the xenograft tumors was also shown in Fig. 7d, further confirming the inhibitory effect of miR-3662 on HCC proliferation.

## Discussion

miRNAs have been revealed as important regulators in cancer progression and metabolic reprogramming^20–22^. Elucidating the association of miRNAs with HCC progression is of great importance to identify novel therapeutic targets and to improve the clinical outcome of this disease. In this study, we demonstrated the relationship between miR-3662 expression level and the clinicopathological features of HCC. We also reported that miR-3662 acted as a tumor suppressor in HCC by directly targeting HIF-1α and ulteriorly inhibiting tumor growth and metabolic reprogramming. miR-3662/HIF-1α axis could be a potential novel molecular target for HCC diagnosis and treatment.

Recent studies reported that miR-3662 dysregulation is involved in human malignancies, including acute myeloid leukemia and lung adenocarcinoma. Maharry et al.^11^ found miR-3662 is not expressed in acute myeloid leukemia, and its overexpression has anti-leukemic effects through targeting IKKβ. Interestingly, Powrózek et al.^12,13^ revealed that miR-3662 expression level is higher in lung adenocarcinoma patients than that in healthy controls. More importantly, their findings suggested that serum miR-3662 may serve as a novel and noninvasive biomarker for the diagnosis of lung adenocarcinoma. Receiver operating characteristic (ROC) curve analysis for miR-3662 precursor allows to distinguish lung adenocarcinoma from squamous cell carcinoma with sensitivity of 71.4%, specificity of 98.5%, and an area under the ROC curve of 0.845^23^. It is intriguing that miR-3662 is downregulated in some types of tumors, but upregulated in others. miRNAs could target many different downstream effectors in different tumors. Some of the target genes promote tumor progression, whereas others exert anti-tumor effects. The downstream regulation network of miRNAs is complex and tissue-specific. Thus, different tumor type could have an unneglectable effect on the role of miR-3662. With regard to the progression of HCC, the specific mechanisms underlying miR-3662 remain poorly known. In the present study, we found that miR-3662 was downregulated in HCC tissues and cell lines, and low expression of miR-3662 was associated with unfavorable clinicopathological features, including large tumor size, tumor multiplicity, advanced Edmondson grade, and high tumor-node-metastasis stage in HCC patients. Furthermore, upregulation of miR-3662 suppressed, while silencing miR-3662 promoted, the Warburg effect and cell proliferation in vitro and tumorigenicity in vivo in HCC cells via HIF-1α. The ERK and JNK signaling pathways were also found to be modulated by miR-3662. These findings indicated that miR-3662 played an important role in the metabolic reprogramming and HCC progression.

Excessively activated in various cancers, the ERK signaling pathway promotes cancer cell proliferation and regulates metabolic reprogramming^24,25^. In addition, tumor growth is closely associated with JNK activation^26,27^. To further provide mechanistic insight into the role of miR-3662 in HCC progression, we examined the impact of miR-3662 expression level on ERK and JNK activation. We observed that the *p*-ERK and *p*-JNK levels were reduced by miR-3662 overexpression. Our data revealed that miR-3662 suppressed the activation of the ERK and JNK signaling pathways, exerting an inhibitory effect on liver cancer growth. Previous studies have established the crucial role of the ERK and JNK signaling in the Warburg effect. The ERK and JNK signaling pathways increase the Warburg effect via multiple mechanisms. ERK phosphorylation causes an increase of PKM2 phosphorylation and upregulates the expression of metabolic genes such as GLUT1 and LDHA expression at protein and mRNA levels. Activation of ERK increases the level of glucose uptake and lactate production^28^. The ERK signaling pathway regulates the ADP-ribosylation factor 6, and promotes cell proliferation and the Warburg effect^29^. Activation of ERK/c-MYC/PFKFB2 also contributes to the Warburg effect^30^. JNK is also in the center of a hub regulating cancer metabolism^31^. JNK interacts with multiple oncogenic pathways including Ras, PI3K/Akt, Raf/ERK, and Src. Altogether this network of genes stabilizes Hifα that in turn, transcriptionally up-regulates many genes encoding glycolytic enzymes^32^. Previous studies also showed that ERK and JNK are upstream of HIF-1α. The ERK pathways converge with PI3K to activate proteins that upregulate the translation of HIF-1α mRNA into protein^33^. In non-small cell lung cancer, HIF-1α is a downstream target of the ERK signaling, and regulates the proliferation and angiogenesis^34^. JNK is an upstream signal of HIF-1α in cancer progression^35,36^. Given that the role of ERK and JNK in the Warburg effect has been clearly reported by previous publications, we did not further explore the role of ERK and JNK in the present study. Our main mechanistic focus is on the downstream effectors of HIF-1α.

HIF-1α is a subunit of the heterodimeric transcription factor HIF-1, and is increased in various human malignancies^37–39^. Overexpressed HIF-1α is closely associated with poor prognosis in HCC^40^. HIF-1α represents a potential therapeutic target in the treatment of liver cancer^33^. HIF-1α dimerizes with HIF-1β, and activates transcription of target genes that play key roles in the metabolic reprogramming of cancer cells^41–43^. HIF-1 activates the transcription of *SLC2A1*, which encodes the glucose transporter GLUT1, and *HK2*, which encodes the first enzyme of glycolytic pathway. PKM2, an enzyme that catalyzes the later step of aerobic glycolysis, as well as LDHA, converting pyruvate to lactate, is also regulated by HIF-1^16,44,45^. Our results showed that ectopic expression of miR-3662 decreased the levels of GLUT1, HK2, PKM2, and LDHA in liver cancer cells after treatment with the hypoxia mimetic CoCl_2_, while silencing miR-3662 increased their expression levels. Reintroduction of HIF-1α rescued miR-3662-mediated reduction in GLUT1, HK2, PKM2, and LDHA expression levels. Thus, the miR-3662/HIF-1α axis exerted its suppressive effect on HCC metabolic reprogramming and cellular proliferation via the regulation of GLUT1, HK2, PKM2, and LDHA expression.

In summary, we found for the first time that miR-3662 was a tumor suppressor in HCC. miR-3662 modulated the Warburg effect, and played an anti-proliferative role in liver cancer. Furthermore, miR-3662 suppressed the Warburg effect and HCC growth via directly targeting HIF-1α after treatment with the hypoxia mimetic CoCl_2_. The ERK and JNK signaling pathways were also regulated by miR-3662. miR-3662 may be a promising biomarker for diagnosis and prognosis, and is a potential therapeutic candidate for HCC.

## Electronic supplementary material

Supplementary Table 1

## References

[1] Torre LA (2015). Global cancer statistics, 2012. CA Cancer J. Clin..

[2] Siegel RL, Miller KD, Jemal A (2017). Cancer statistics, 2017. CA Cancer J. Clin..

[3] Bosetti C, Turati F, La VC (2014). Hepatocellular carcinoma epidemiology. Best. Pract. Res. Clin. Gastroenterol..

[4] Miller KD (2016). Cancer treatment and survivorship statistics, 2016. CA Cancer J. Clin..

[5] Pavlova NN, Thompson CB (2016). The emerging hallmarks of cancer metabolism. Cell. Metab..

[6] Warburg O (1956). On the origin of cancer cells. Science.

[7] Vander Heiden MG, Cantley LC, Thompson CB (2009). Understanding the Warburg effect: the metabolic requirements of cell proliferation. Science.

[8] Hay N (2016). Reprogramming glucose metabolism in cancer: can it be exploited for cancer therapy?. Nat. Rev. Cancer.

[9] Ha M, Kim VN (2014). Regulation of microRNA biogenesis. Nat. Rev. Mol. Cell Biol..

[10] Zhang LF, Jiang S, Liu MF (2017). MicroRNA regulation and analytical methods in cancer cell metabolism. Cell Mol. Life. Sci..

[11] Maharry SE (2016). Dissection of the major hematopoietic quantitative trait locus in chromosome 6q23.3 identifies miR-3662 as a player in hematopoiesis and acute myeloid leukemia. Cancer Discov..

[12] Powrozek T, Mlak R, Dziedzic M, Malecka-Massalska T, Sagan D (2017). Analysis of primary-miRNA-3662 and its mature form may improve detection of the lung adenocarcinoma. J. Cancer Res. Clin. Oncol..

[13] Powrozek T (2015). Plasma circulating microRNA-944 and microRNA-3662 as potential histologic type-specific early lung cancer biomarkers. Transl. Res..

[14] Vondran FW (2008). Isolation of primary human hepatocytes after partial hepatectomy: criteria for identification of the most promising liver specimen. Artif. Organs.

[15] Wang M (2017). HIF-1α promoted vasculogenic mimicry formation in hepatocellular carcinoma through LOXL2 up-regulation in hypoxic tumor microenvironment. J. Exp. Clin. Cancer Res..

[16] Semenza GL (2010). HIF-1: upstream and downstream of cancer metabolism. Curr. Opin. Genet. Dev..

[17] Li B (2017). Mutual regulation of miR-199a-5p and HIF-1alpha modulates the Warburg effect in hepatocellular carcinoma. J. Cancer.

[18] Kai AK (2016). Down-regulation of TIMP2 by HIF-1alpha/miR-210/HIF-3alpha regulatory feedback circuit enhances cancer metastasis in hepatocellular carcinoma. Hepatology.

[19] Xue TM (2015). Clinicopathological significance of microRNA-20b expression in hepatocellular carcinoma and regulation of HIF-1alpha and VEGF effect on cell biological behaviour. Dis. Markers.

[20] Li L (2017). miR-30a-5p suppresses breast tumor growth and metastasis through inhibition of LDHA-mediated Warburg effect. Cancer Lett..

[21] Ma X (2014). Lin28/let-7 axis regulates aerobic glycolysis and cancer progression via PDK1. Nat. Commun..

[22] Guo X (2017). miR-181d and c-myc-mediated inhibition of CRY2 and FBXL3 reprograms metabolism in colorectal cancer. Cell Death Dis..

[23] Powrozek T (2017). The diagnostic role of plasma circulating precursors of miRNA-944 and miRNA-3662 for non-small cell lung cancer detection. Pathol. Res. Pract..

[24] Cao Y (2016). Prohibitin overexpression predicts poor prognosis and promotes cell proliferation and invasion through ERK pathway activation in gallbladder cancer. J. Exp. Clin. Cancer Res..

[25] Samatar AA, Poulikakos PI (2014). Targeting RAS-ERK signalling in cancer: promises and challenges. Nat. Rev. Drug. Discov..

[26] Xu Z (2017). Sophoridine induces apoptosis and S phase arrest via ROS-dependent JNK and ERK activation in human pancreatic cancer cells. J. Exp. Clin. Cancer Res..

[27] Lin, S., et al. Melatonin promotes sorafenib-induced apoptosis through synergistic activation of JNK/c-jun pathway in human hepatocellular carcinoma. *J. Pineal. Res*. **62**, 2017. 10.1111/jpi.12398 28178378

[28] Lee KM (2015). ECM1 promotes the Warburg effect through EGF-mediated activation of PKM2. Cell Signal..

[29] Liang C (2017). ARF6, induced by mutant Kras, promotes proliferation and Warburg effect in pancreatic cancer. Cancer Lett..

[30] Zhao SJ (2018). SLIT2/ROBO1 axis contributes to the Warburg effect in osteosarcoma through activation of SRC/ERK/c-MYC/PFKFB2 pathway. Cell Death Dis..

[31] Papa S, Bubici C (2016). Linking apoptosis to cancer metabolism: Another missing piece of JuNK. Mol. Cell Oncol..

[32] Wang CW, Purkayastha A, Jones KT, Thaker SK, Banerjee U (2016). In vivo genetic dissection of tumor growth and the Warburg effect. eLife.

[33] Lin D, Wu J (2015). Hypoxia inducible factor in hepatocellular carcinoma: a therapeutic target. World J. Gastroenterol..

[34] Wan J, Wu W (2016). Hyperthermia induced HIF-1a expression of lung cancer through AKT and ERK signaling pathways. J. Exp. Clin. Cancer Res..

[35] Yang S, Qiang L, Sample A, Shah P, He YY (2017). NF-κB signaling activation induced by chloroquine requires autophagosome, p62 protein, and c-Jun N-terminal Kinase (JNK) signaling and promotes tumor cell resistanc*e*. J. Biol. Chem..

[36] Shi L, Zhang G, Zheng Z, Lu B, Ji L (2017). Andrographolide reduced VEGFA expression in hepatoma cancer cells by inactivating HIF-1α: The involvement of JNK and MTA1/HDCA. Chem. Biol. Interact..

[37] Palazon A (2017). An HIF-1alpha/VEGF-A axis in cytotoxic T cells regulates tumor progression. Cancer Cell.

[38] Miroshnikova YA (2016). Tissue mechanics promote IDH1-dependent HIF1alpha-tenascin C feedback to regulate glioblastoma aggression. Nat. Cell Biol..

[39] Bruno T (2017). Che-1 sustains hypoxic response of colorectal cancer cells by affecting Hif-1α stabilization. J. Exp. Clin. Cancer Res..

[40] Liu L (2016). The impact of high co-expression of Sp1 and HIF1alpha on prognosis of patients with hepatocellular cancer. Oncol. Lett..

[41] Rankin EB, Giaccia AJ (2016). Hypoxic control of metastasis. Science.

[42] Shukla SK (2017). MUC1 and HIF-1alpha signaling crosstalk induces anabolic glucose metabolism to impart gemcitabine resistance to pancreatic cancer. Cancer Cell.

[43] Li H, Rokavec M, Jiang L, Horst D, Hermeking H (2017). Antagonistic effects of p53 and HIF1A on microRNA-34a regulation of PPP1R11 and STAT3 and hypoxia-induced epithelial to mesenchymal transition in colorectal cancer cells. Gastroenterology.

[44] Massari F (2016). Metabolic phenotype of bladder cancer. Cancer Treat. Rev..

[45] Azoitei N (2016). PKM2 promotes tumor angiogenesis by regulating HIF-1alpha through NF-kappaB activation. Mol. Cancer.

